# Childbirth at home and associated factors in Ethiopia: a systematic review and meta-analysis

**DOI:** 10.1186/s13690-021-00569-5

**Published:** 2021-04-13

**Authors:** Asteray Assmie Ayenew, Azezu Asres Nigussie, Biruk Ferede Zewdu

**Affiliations:** 1 Department of Midwifery, School of Health Sciences, College of Medicine and Health Sciences, Bahir Dar University, Bahir Dar, Ethiopia; 2 Department of Orthopedics, School of Medicine, College of Medicine and Health Sciences, Bahir Dar University, Bahir Dar, Ethiopia

**Keywords:** Childbirth at home, Maternal mortality, Systematic review and meta-analysis, Ethiopia

## Abstract

**Background:**

Maternal mortality remains a major challenge to health systems worldwide. Although most pregnancies and births are uneventful, approximately 15% of all pregnant women develop potentially life-threatening complications. Childbirth at home in this context can be acutely threatening, particularly in developing countries where emergency care and transportation are less available. Therefore, this systematic review and meta-analysis aimed to assess the prevalence of home childbirth and its associated factors among women in Ethiopia at their last childbirth.

**Method:**

For this review, we used the standard PRISMA checklist guideline. This search included all published and unpublished observational studies written only in English language and conducted in Ethiopia. PubMed/Medline, Hinari, EMBASE, Google Scholar, Science Direct, Scopus, Web of Science (WoS), ProQuest, Cochrane Library, African Journals Online, Ethiopian’s university research repository online library were used. Based on the adapted PICO principles, different search terms were applied to achieve and access the essential articles from February 1–30, 2020. The overall selected search results were 40 studies. Microsoft Excel was used for data extraction and Stata version 11.0 (Stata Corporation, College Station, Texas, USA) for data analysis. The quality of individual studies was appraised by using the Joanna Briggs Institute (JBI) quality appraisal checklist. The heterogeneity of the studies was assessed by the Cochrane Q and I2 test. With the evidence of heterogeneity, subgroup analysis and sensitivity analysis were computed. The pooled prevalence of childbirth at home and the odds ratio (OR) with a 95% confidence interval was presented using forest plots.

**Result:**

Seventy-one thousand seven hundred twenty-four (71, 724) mothers who gave at least one birth were recruited in this study. The estimated prevalence of childbirth at home in Ethiopia was 66.7% (95%CI: 61.56–71.92, I2 = 98.8%, *p*-value < 0.001). Being from a rural area (adjusted odds ratio (AOR) 6.48, 95% confidence interval (CI): 3.48–12.07), being uneducated (AOR = 5.90, 95% CI: 4.42–7.88), not pursuing antenatal (ANC) visits at all (AOR = 4.57(95% CI: 2.42–8.64), having 1–3 ANC visits only (AOR = 4.28, 95% CI: 3.8–8.26), no birth preparedness and complication readiness plan (AOR = 5.60, 95% CI: 6.68–8.25), no media access (AOR = 3.46, 95% CI: 2.27–5.27), having poor knowledge of obstetric complications (AOR = 4.16: 95% CI: 2.84–6.09), and walking distance more than 2 hours to reach the nearest health facility (AOR = 5.12, 95% CI: 2.94–8.93) were the factors associated with giving childbirth at home.

**Conclusion:**

The pooled prevalence of childbirth at home was high in Ethiopia. Being from a rural area, being uneducated, not pursuing ANC visits at all, having 1–3 ANC visits only, no media access, having poor knowledge of obstetric complications, not having a birth preparedness and complication readiness plan, and walking time greater than 2 hours to reach the nearest health facility increased the probability of childbirth at home in Ethiopia.

## Background

Childbirth at home is a practice of giving birth in a nonclinical setting that takes place in residence than in the birth center or hospitals, which could be unhygienic, unsupervised, and when interventions are needed it is usually late [1]. It plays a vital role as the place to maternal death occurrence due to obstetric complications like hemorrhage, hypertensive disorders, sepsis, abortion, embolism, and all other direct causes of death [2].

Despite improvements, over 25 years between 1990 and 2015, a total of 13.6 million women have died due to maternal causes. Developing regions account for approximately 99% of the estimated global maternal deaths in 2015, with sub-Saharan Africa alone accounting for roughly 66%, followed by Southern Asia [3]. Moreover, in sub-Saharan Africa, a woman’s risk of dying from preventable obstetric during her lifetime is 1 in 22 as compared to 1 in 7300 in developed countries [4].

Evidences showed that about 66% of all maternal deaths worldwide and over 50% in developing countries were directly related to unsafe delivery practices [5]. Although it is known that skilled health professionals are key actors to reduce maternal mortality by preventing and managing complications during pregnancy and childbirth, still a number of women are died due to giving birth without the attendance of skilled health workers [6–8]. According to WHO, all women needs access to health services such as prenatal visits, skilled birth attendant and postnatal care visits [7]. Despite, a high proportion of women had received antenatal care services, only one in three women is utilized institutional delivery in developing countries [9]. Delivery care practice differs with respect to residence, culture, availability, and accessibility of the health care services [10]. Increasing institutional delivery is the central goal of safe motherhood and child survival movements [11].

Ethiopia’s maternal mortality ratio has decreased from an estimated 678 deaths per 100,000 live births in 2011, accounting for 412 deaths per 100,000 live births [12] in 2016, it remains unacceptably high.

A vast majority of maternal deaths are due to preventable direct obstetric causes that can be detected and managed early during antenatal care (ANC) and intrapartum period by existing and well-known medical interventions [13]. If home delivery is not conducted by professionals; it increases the risk of infection, postpartum hemorrhage (PPH), and transmission of HIV/AIDS to relatives or traditional birth attendants, who conduct deliveries without protective equipment’s [14]. The burden of home childbirth, mainly that of unattended delivery, is not only limited to maternal health problems, but it also ends up with perinatal and neonatal morbidity and mortality [15].

Ethiopia was a country where a low proportion of reproductive-age women visit skilled providers during pregnancy and childbirth [16]. Moreover, a great majority (74%) of women nationwide gave birth at home although, 62% of women attend antenatal care [17].

Deliveries in health facilities are associated with mortality reductions for both newborns and mothers [18, 19]. Access to skilled care at every birth and facility with the capacity to manage emergency, obstetric, and newborn complications is a key strategy to reduce maternal and newborn mortality [20]. However, most deliveries in developing countries occur at home without skilled birth attendants [4, 21].

Various studies conducted in different developing countries and in different parts of Ethiopia revealed different factors associated with home birth. Among the identified factors is lack of access to health facilities, increased parity, counseling services obtained during ANC visits, maternal age, age at first pregnancy, age at first marriage, absence of previous obstetric complications, health care providers’ behavior, quality of ANC services, and decision-makers on the place of delivery [22–25].

Additionally, studies revealed that women living in rural areas and distant from health facilities tend to give birth at home [26–28]. Maternal education [29, 30], knowledge of pregnancy and pregnancy-related complications are also an important factor that affects attitude, intension, and behavior towards health service utilization [31–33]. Thus, The poor knowledge they have about dangerous signs of pregnancy, and home childbirth the more they tend to give birth at home [32, 34]. Although most pregnancy and delivery related complications cannot be predicted, high quality antenatal care (ANC) and receiving counseling on birth preparedness during antenatal care appeared to strongly influence women’s use of skilled care during delivery [32, 33, 35]. A strong association was also shown to exist between the quality of care obtained during pregnancy and home childbirth [26, 29]. Moreover, lack of money, lack of transport, sudden onset of labor, lack of privacy, geographical location, perception of poor quality of health services, tradition, cultures, and the pattern of decision-making power within the household were key determinants of home childbirth [36–38].

The main adverse outcomes in patients admitted due to obstetric complications after home childbirth were Postpartum hemorrhage in 48% of patients (of which primary 61.2% and secondary in 38.8% patients) followed by retained placenta/placental tissues in 26% of women. Three women died out of a total of 261 admitted patients (1.1%) due to puerperal sepsis within a few hours of admission which is a very high frequency of maternal deaths among patients who delivered at home [2]. About 73% of all maternal deaths were due to direct obstetric causes like hemorrhage, hypertensive disorders, sepsis, and abortion, but the top three leading causes are hemorrhage, hypertensive disorders, and sepsis which are responsible for more than 50% of maternal deaths worldwide [39]. The highest number of maternal deaths occurs on the first day after delivery highlighting the critical need for institutional service utilization during delivery [40].

In Ethiopia, despite the launching of the Health Extension Program and improving access to healthcare throughout the country [41], the prevalence of childbirth at home is stagnant so far, ranging from 20% in Amhara region [42] to 92.5% in Afar [43]. Therefore, the aim of this systematic review and meta-analysis was to estimate the pooled prevalence of home childbirth and associated factors among women in Ethiopia at their last childbirth.

## Methods

This systematic review and meta-analysis were conducted to estimate the prevalence of home childbirth and associated factors among women in Ethiopia at their last childbirth. We used the Preferred Reporting Items for Systematic Reviews and Meta-Analyses (PRISMA) checklist guideline [44].

### Searching strategy

First, the PROSPERO database and database of abstracts of reviews of effects (DARE) (http://www.library.UCSF.edu) were searched to check whether published and/or ongoing projects exist related to the topic. The literature search strategy, selection of studies, data extraction, and result reporting were done in accordance with (PRISMA) guidelines [45]. Searching terms were based on adapted PICO principles to search through the above-listed databases to access the relevant articles. The search string was developed using “AND” and “OR” Boolean operators. Articles accessed in the PubMed/Medline, Hinari, EMBASE, Google Scholar, Science Direct, Scopus, WoS, ProQuest, Cochrane Library, African Journals Online, and online university repositories (University of Gondar and Addis Ababa University) was used) were considered in this systematic review and meta-analysis (Table 1). Different MeSH terms and search engines including “home childbirth” OR “giving birth at home” AND “factors,” OR “determinants” AND related in Ethiopia.

**Table 1:** Search for PubMed and Google Scholar databases to assess home childbirth in Ethiopia

Databases	Searching terms	Number of studies
PubMed	[(“home delivery”[All Fields] OR “home birth” [MeSH Terms]) AND (“rural mothers” [All Fields]) AND (“delivery place preference” [All Fields]) OR (“home childbirth”[All Fields] AND “socio-demographic factors”[All Fields] AND “birth”[All Fields] AND “delivery”[All Fields]) OR “childbirth”[All Fields] OR (“mothers”[All Fields] AND “reproductive age women”[All Fields]) OR “traditional birth attendants “[All Fields]) OR ((“mothers”[MeSH Terms] OR “mothers”[All Fields] OR “reproductive age mothers”[All Fields]) AND factors [All Fields] AND (“ethiopia”[MeSH Terms] OR “ethiopia”[All Fields])]	485
Google scholar	“Home birth” or “giving birth at home” or “home delivery” and “factors,” or “determinants” AND Ethiopia	227
From other databases		68
Total retrieved articles		780
Number of included studies		40

### Eligibility criteria

#### Inclusion criteria

Study Design: All observational studies reported the prevalence of home childbirth and/or associated factors were included.

Language: English language literature and research articles were included.

Publication: Both unpublished and published research articles were considered.

Searching date: Articles searched from February 1–30, 2020 were included.

#### Exclusion criteria

Duplicated studies, articles without full text and abstract, anonymous reports, qualitative studies, and editorial reports were excluded.

### Data extraction and quality assessment

The Standard Microsoft Excel spreadsheet was used to export data from online databases. Three authors independently extracted and reviewed the articles included in this study. Any disagreement was handled by the fourth reviewer, and a consensus was reached through discussion between authors. Two investigators assessed the quality of studies using the JBI quality appraisal criteria appraisal checklist adapted for cross-sectional, case-control, and cohort studies [46]. Studies considered low risk whenever fitted to 50% and or above quality assessment score.

### Outcome of measurement

The prevalence of childbirth at home was the main outcome of the study. The prevalence and adjusted odds ratios were calculated for risk factors reported in the study.

Home childbirth: When a mother gave birth at her home or others’ home (neighbor, relatives, or family) or when a birth takes place outside of health institutions [47].

Knowledgeable: was considered a woman who scored above the mean of obstetric complications knowledge assessment questions and otherwise they were considered as having poor knowledge [48].

### Statistical analysis

The Microsoft Excel (2016) was used for the data extraction and STATA version 11 software for used for analysis. As the test statistic showed significant heterogeneity among studies (I^2^ = 98.8%, *p* < 0.05) the Random-effects model was used to estimate the DerSimonian and Laird’s pooled effect [49]. The funnel plot and Egger’s regression test were conducted to check potential publication bias [50, 51]. The Cochrane Q test and I^2^ were used to assess the heterogeneity of the study. The values of 25, 50, and 75% were declared as low, moderate, and high heterogeneity respectively [52, 53]. Hence, there was high heterogeneity within studies; the random effect model [54] was used to compute the pooled prevalence of home childbirth. Furthermore, due to the presence of heterogeneity within studies, subgroup and sensitivity analysis was computed. Moreover, the estimated pooled prevalence rate was reported with a 95% confidence interval (CI), and *P*-value < 0.05 was considered statistically significant.

## Results

### Characteristics of the included studies

We retrieved 755 studies from the PubMed/MEDLINE, Google Scholar, HINARI, EMBASE, Science Direct, Scopus, WoS, ProQuest, Cochrane library, African Journals, and online university repository research articles. After duplicates were expunged, 324 studies remained.

**Fig. 1:**
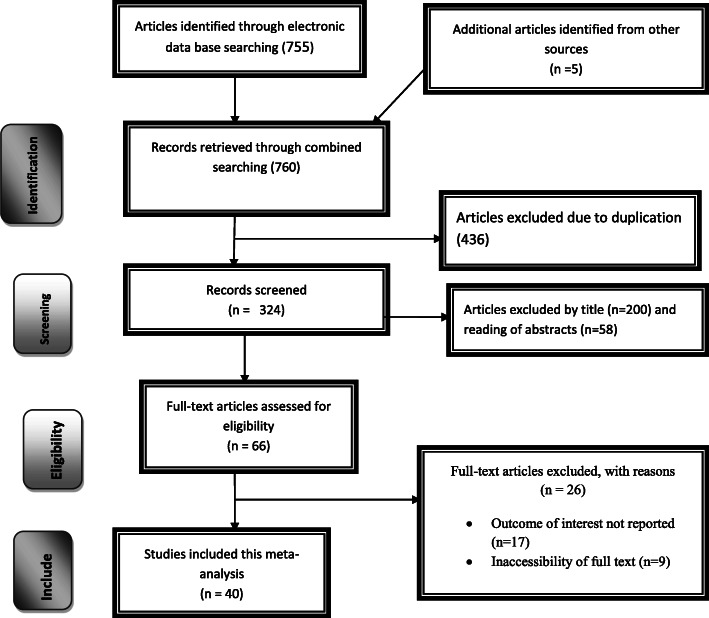
PRISMA 2009 Flow diagram for identification and selection of articles for inclusion in this review

Out of the remaining 324 articles, 258 articles were excluded after review of their titles and abstracts. As a result, for inclusion criteria, 66 full-text articles were accessed and assessed, which resulted in the further exclusion of 26 articles. Out of these, 17 studies were excluded due to the outcome of interest were not reported, and 9 of them were excluded due to inaccessibility of the full text. As a result, 40 studies were met the inclusion criteria to undergo the final systematic review and meta-analysis (Fig. 1).

In this review, 40 relevant studies with a sample size of 71, 724 were included. Studies were conducted from different regions of Ethiopia (Amhara, Oromia, SNNPR (South Nation Nationalities people and representatives), Afar, and Tigray) (Table 2). Among the included, 35 articles were cross-sectional, 3 cohorts, and 2 were case-control studies in its design.

**Table 2:** Descriptive summary of forty included studies in the systematic review and meta-analysis (*n* = 40)

Author (year)	study design (setting)	Sample size	Study region	Prevalence (%)
Yilkal M.et al.(2015) [22]	Community based Cross-sectional	499	SNNPR	75.3
Hinsermu B.et al.(2014) [42]	Institutional based Cohort study	422	Amhara	20
Shabeza A. et al.(2016) [55]	Community based Cross-sectional	276	Amhara	49.3
Abeba D.et al.(2017) [56]	Community based Cross-sectional	295	SNNPR	45.8
Habtamu K. et al.(2016) [57]	Community based Cross-sectional	518	Amhara	25.3
Solomom S. et al.(2013) [58]	Community based Cross-sectional	909	SNNPR	78
Anteneh A.et al.(2013) [59]	Community based Cross-sectional	2390	SNNPR	62.2
Getiye D. et al.(2014) [60]	Community based Cross-sectional	453	Amhara	75.3
Bedlu K. et al.(2015) [61]	Community based Cross-sectional	477	SNNPR	67.7
Zemenu T.et al. (2016) [62]	Community based Cross-sectional	34,348	SNNPR	73.4
Melese S.et al. (2017) [63]	Institutional based Cohort study	554	SNNPR	73.5
Gistane A. et al. (2012) [64]	Community based Cross- sectional	481	Oromia	78
Momina A.et al. (2016) [65]	Community based cross-sectional	318	Afar	74
Teklemariam G.et al. (2016) [66]	Community based Cross -sectional	285	SNNPR	79
Tilahun W.et al. (2018) [67]	Community based cross-sectional	576	Amhara	55.2
Resom T.et al. (2014) [68]	Community based case control	275	Tigray	N/A
Fantu A.at al (2018) [69]	Institutional based Case control	324	Amhara	N/A
Abebe A.et al. [70]	Community based Cross-sectional	772	SNNPR	45.3
Dejene K. et al.(2018) [71]	Community based Cross –sectional	507	SNNPR	68
Mednanit G. et al.(2011) [43]	Community based cross-sectional	478	Afar	92.5
Arya M .et al. (2016) [72]	Community based cross sectional	528	Amhara	52.7
Alemayehu S.et al. [24]	Community based cross sectional	371	Amhara	87.9
Abdella A.et al. [73]	Institutional based cross sectional	855	Oromia	87.7
Feleke H. et al. [74]	Community based cross sectional	845	SNNPR	69
Desta H. et al. [75]	Community based cross sectional	485	Amhara	69.5
Deneke D. et al. [76]	Community based cross sectional	552	SNNPR	73.6
Mihiretu A. et al. [77]	Community based cross sectional	957	SNNPR	62
Masresha A.et al. [78]	Community based cross sectional	411	Amhara	74
Melaku F. et al. [79]	Community based cross sectional	6641	Amhara	83.4
Nigussu T. et al. [80]	Community based cross sectional	777	Amhara	79
Melese G. et al. [81]	Community based cross sectional	748	Amhara	55
Mohhamed A. et al. [82]	Community based cross sectional	2009	Afar	89
Yeshalem M. et al. [83]	Community based cross sectional	763	Amhara	82.7
Bayu H. et al. [84]	Institutional based Cohort study	522	Tigray	28.8
Tewodros E. et al. [85]	Community based cross sectional	623	Afar	39.5
Sewnet K. et al. [86]	Community based cross sectional	674	Amhara	66
Alemayehu S. et al. [24]	Community based cross sectional	371	Amhara	87.9
Yabyo H. et al. [87]	Community based cross sectional	484	Amhara	88.3
Addis A.et al. [88]	Community based cross sectional	506	Oromia	81.8
Seifu H. et al. [89]	Community based cross sectional	4949	Tigray	84.4

*SNNPR* Southern Nation Nationality and Peoples Representatives

### Prevalence of childbirth at home in Ethiopia

**Fig. 2:**
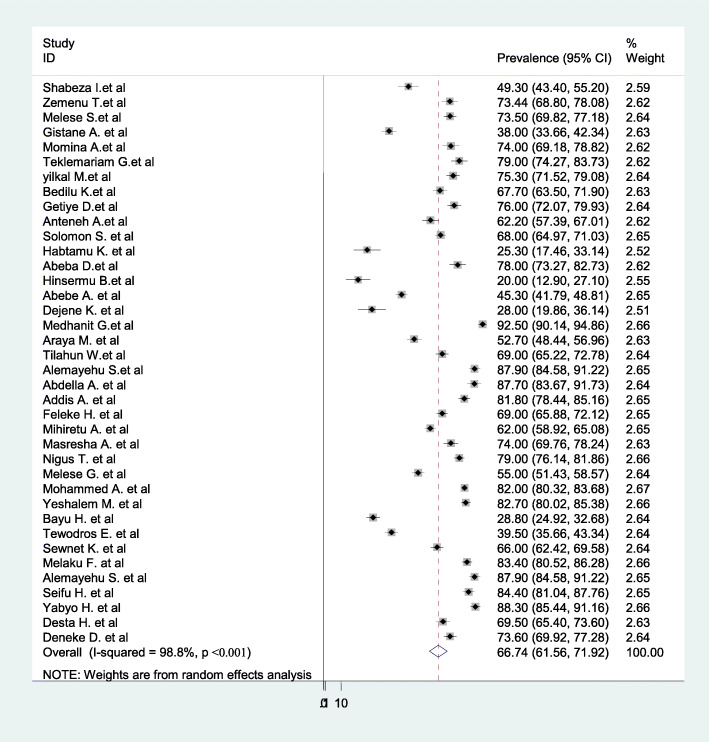
Forest Plot for the pooled prevalence of childbirth at home in Ethiopia

We excluded 2 case-control studies on the prevalence estimation (Fantu A.at al (2018) [69] and Resom T.et al. (2014) [68]). The overall prevalence of home childbirth is presented with a forest plot (Fig. 2). Therefore, the pooled prevalence of home childbirth in Ethiopia was 66.7% (95%CI: 61.56–71.92, I^2^ = 98.8%, *p*-value < 0.001).

### Heterogeneity and publication bias

**Fig. 3:**
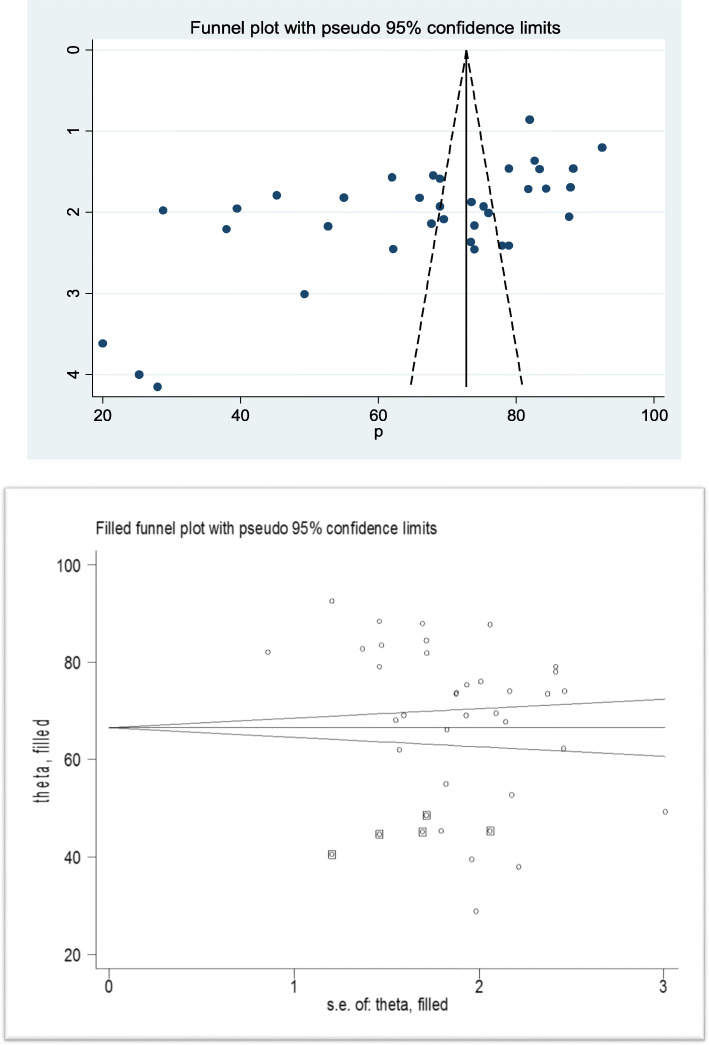
**a** Funnel plot test for publication bias for childbirth at home in Ethiopia. **b** Funnel plot test of trim filled analysis for childbirth at home in Ethiopia

In this meta-analysis, we executed heterogeneity within the included studies, indicating the presence of considerable heterogeneity (I^2^ = 98.8%, *p*-value < 0.001). We also assessed the presence of publication bias by using Egger’s test, which suggests the presence of publication bias (0.001). As a result, trim and fill analysis was conducted to overcome the publication bias. After three studies were filled, thirty five studies were enrolled and computed through the trim and fill analysis with a pooled prevalence of 66.49% (95% CI: 61.01–71.93) using a random effect model (Fig. 3a and b).

### Sensitivity analysis

**Fig. 4:**
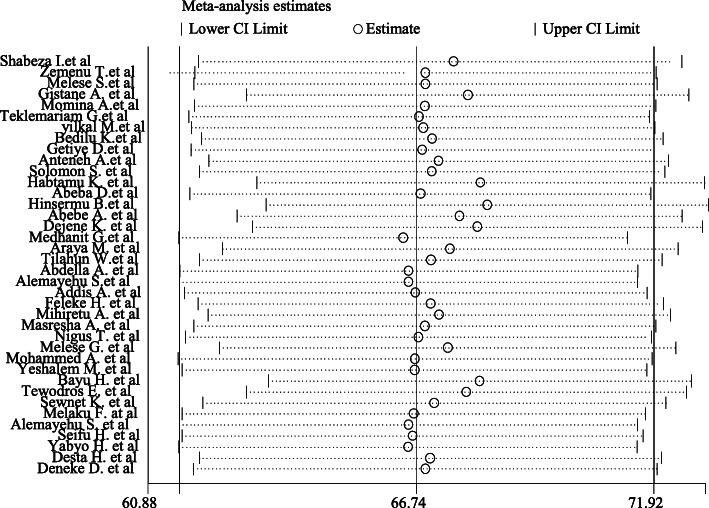
Sensitivity analysis of the pooled prevalence of giving birth at home in Ethiopia

In this meta-analysis, to investigate the potential source of heterogeneity observed in pooled prevalence of childbirth at home, a leave-one-out sensitivity analysis was executed and suggesting that our findings was not dependent on a single study. Thus, the point estimate of its omitted analysis lies within the confidence interval of the combined analysis (Fig. 4).

### Subgroup analysis

**Fig. 5:**
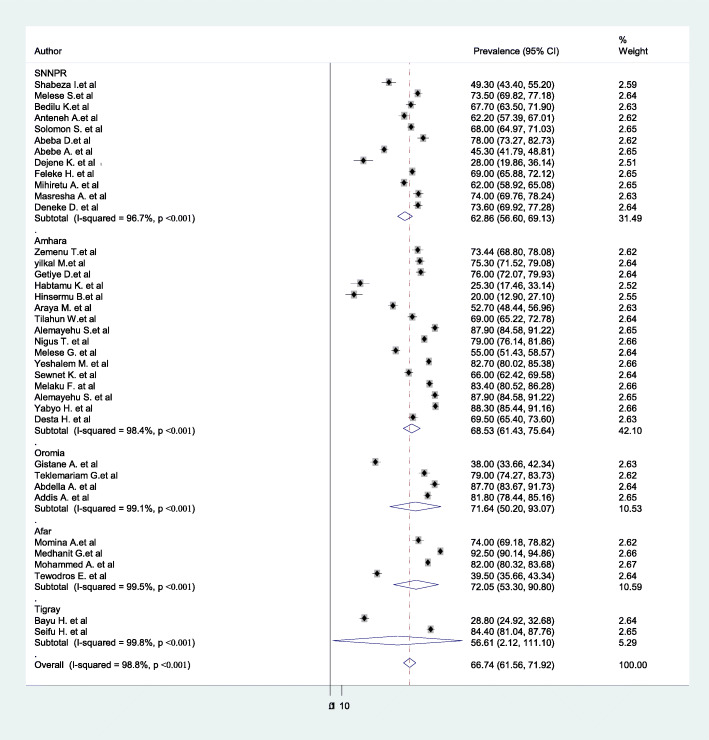
Forest plot of the subgroup analysis based on the study area (region)

**Fig. 6:**
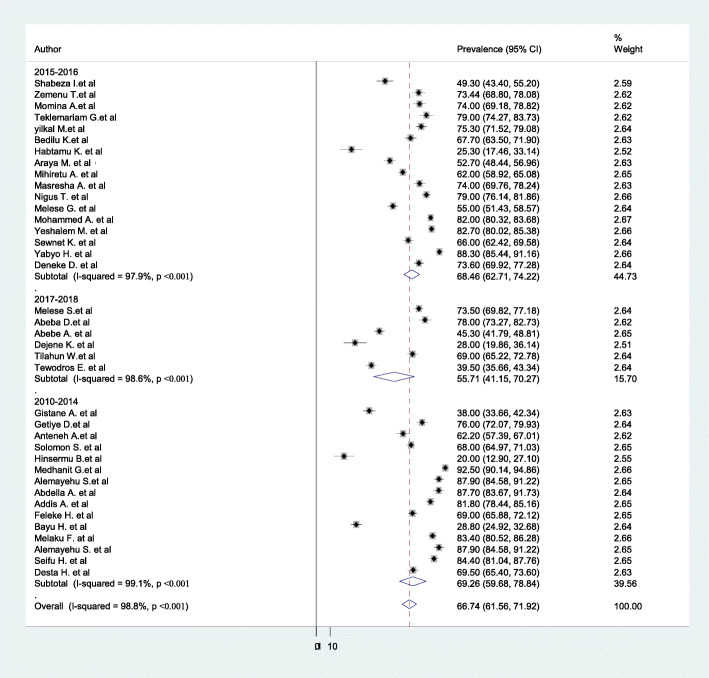
Forest plot of the subgroup analysis based on year of publication

In this meta-analysis, we executed heterogeneity within the included studies, indicating the presence of considerable heterogeneity (I^2^ = 98.8%, *p* < 0.001). Due to considerable heterogeneity subgroup analysis was done by study regions and year of the study. Based on this, the highest prevalence of home childbirth was in Afar region 72.1% (95%CI: 53.30–90.80, I^2^ = 99.5, *P* < 0.001), and the lowest was in Tigray region 56.6% (95%CI: 2.21–111.10, I^2^ = 99.8, *P* < 0.001) (Fig. 5). Based on year of publication the highest prevalence was between 2010 & 2014 (69.3%; 95%CI: 59.68–78.84, I^2^ = 99.1, P < 0.001), and the lowest was between 2017& 2018 (55.7%; 95%CI: 41.15–70.27, I^2^ = 98.6, *P* < 0.001) (Fig. 6).

### Determinants of home childbirth

In this systematic review and meta-analysis, we have examined the associations of factors (Women’s residency, women’s level of education, not pursuing ANC visits at all, having 1–3 ANC visits only, no media access, having poor knowledge of obstetric complications, have no birth preparedness and complication readiness plan, and walking time greater than 2 hours to reach the nearest health facility) with childbirth at home.

**Fig. 7:**
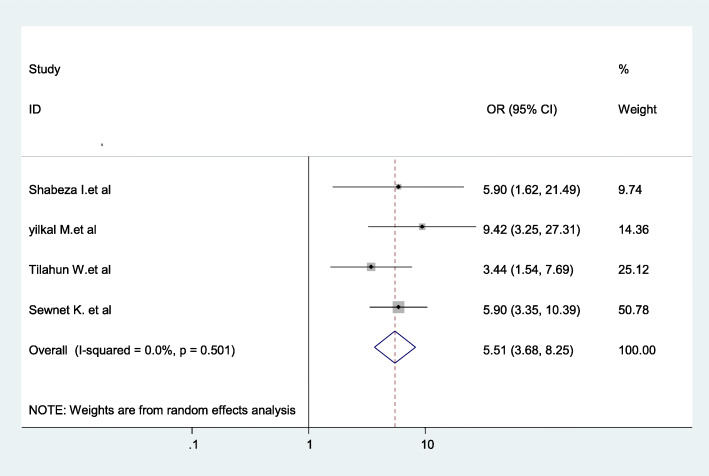
Association between birth preparedness and complication readiness plan and childbirth at home in Ethiopia

The findings of the review indicated a significant association between birth preparedness and complication readiness plan and giving birth at home. Women who had no birth preparedness and complication readiness plan were 5.51 times more likely to give childbirth at home as compared to those who had birth preparedness and complication readiness plan during pregnancy (AOR = 5.51; 95% CI: 6.68–8.25) (Fig. 7).

**Fig. 8:**
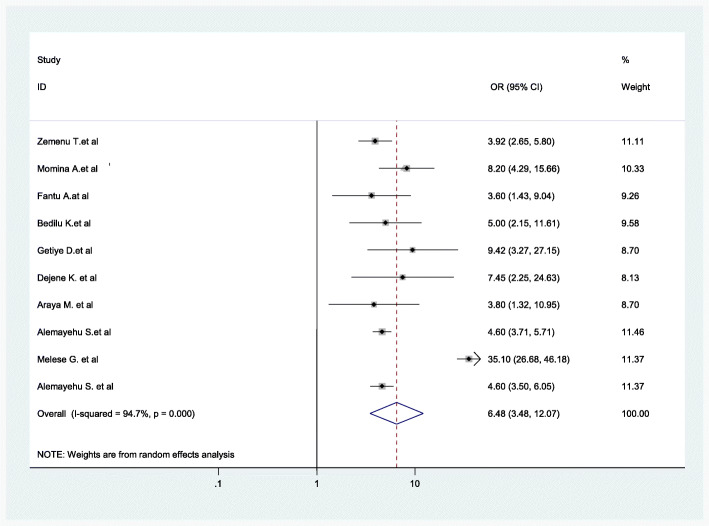
Association between women’s residency and childbirth at home in Ethiopia

Women’s residency (as defined as rural and urban) was significantly associated with home childbirth. Women from rural areas were more likely to give birth at home than those (women) from urban areas (AOR = 6.48, 95% CI: 3.48–12.07) (Fig. 8).

**Fig. 9:**
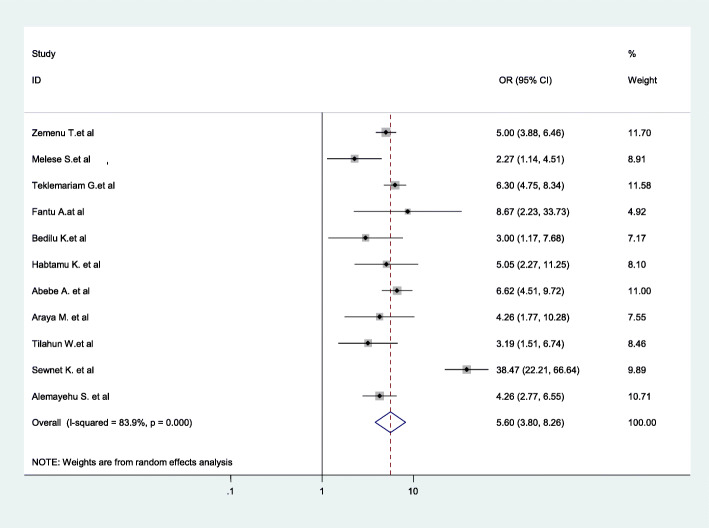
Association between frequencies of ANC follow-up and childbirth at home in Ethiopia

Women who had 1–3 ANC visits only were 5.60 times more likely to give birth at home as compared to women who had 4 or more ANC visits (AOR = 5.60, 95% CI: 3.8–8.26) (Fig. 9).

**Fig. 10:**
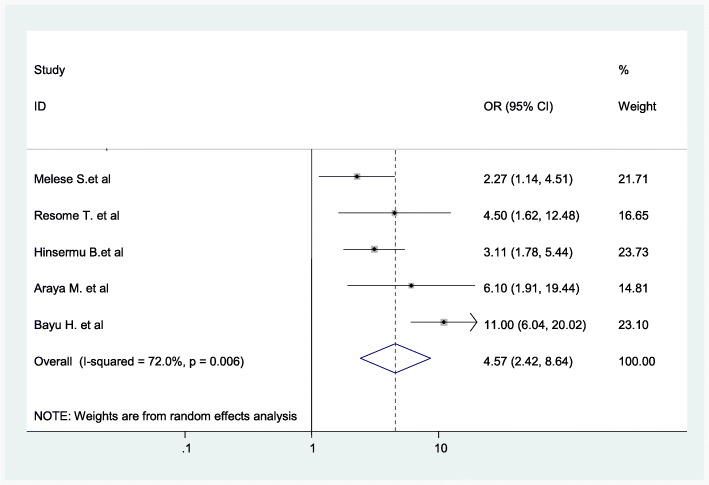
Associations between women of not pursuing ANC at all and childbirth at home

Women who did not have ANC follow-up at all were 4.57 times (AOR = 4.57; 95% CI: 2.42–8.64) more likely to give birth at home as compared to those women who had 4 or more ANC visits (Fig. 10).

**Fig. 11:**
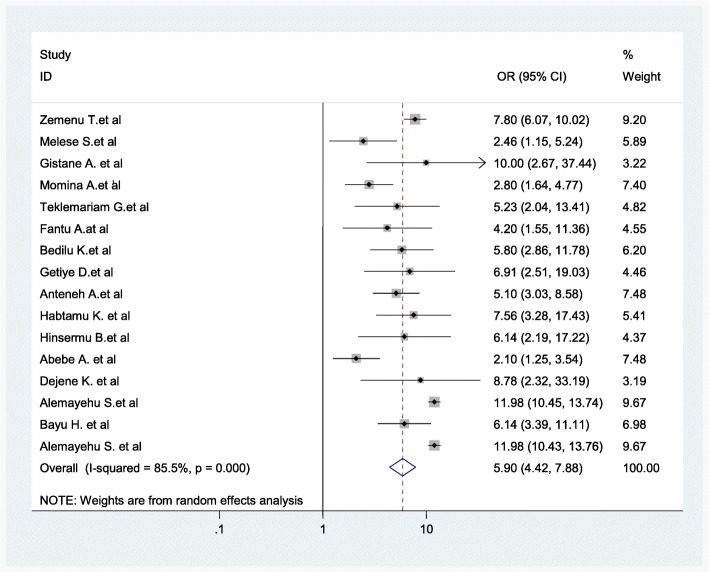
Association between women’s educational level and childbirth at home in Ethiopia

Women who had no formal education were nearly 5.90 times more likely to give birth at home as compared to women who had primary education and above (AOR = 5.90; 95% CI: 4.42–7.88) (Fig. 11).

**Fig. 12:**
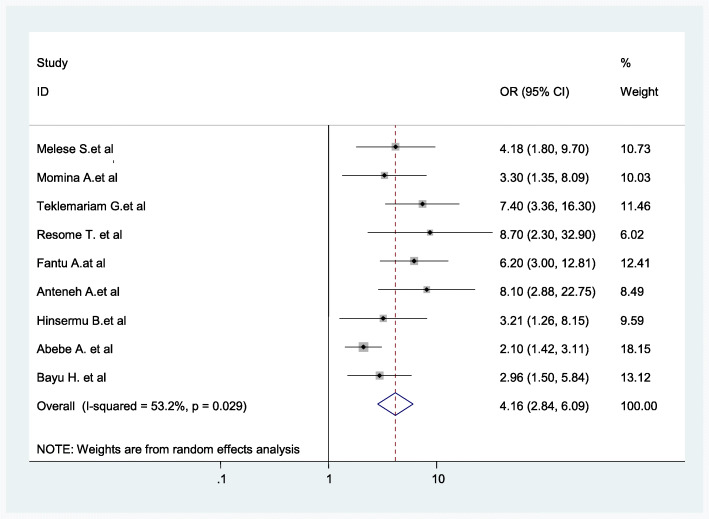
Association between woman’s knowledge level of obstetric complications and childbirth at home

The level of women’s knowledge of obstetric complications was significantly associated with home childbirth. Women who had poor knowledge of obstetric complications were more likely to give birth at home as compared to women who had adequate knowledge of obstetric complications (AOR = 4.16; 95% CI: 2.84–6.09) (Fig. 12).

**Fig. 13:**
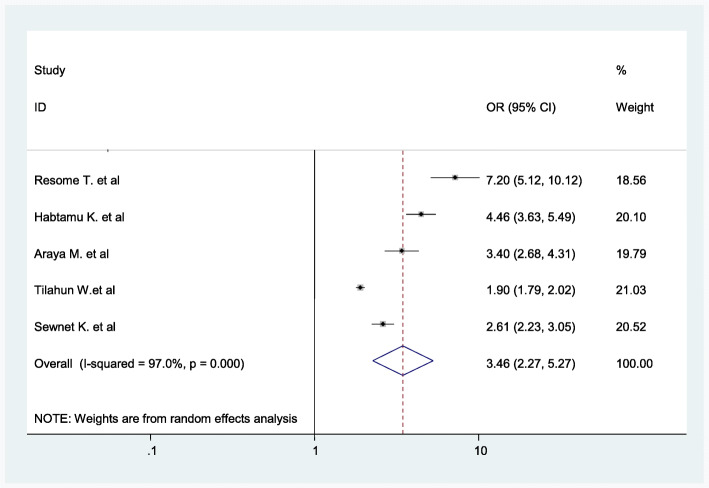
Association between no media access and childbirth at home in Ethiopia

The findings of the review indicated a significant association between women’s access to media and home birth. Women who have no access to media were nearly 3.46 times more likely to give birth at home as compared to women who had access to electronic media (radio/television) (AOR = 3.46; 95% CI: 2.27–5.27)(Fig. 13).

**Fig. 14:**
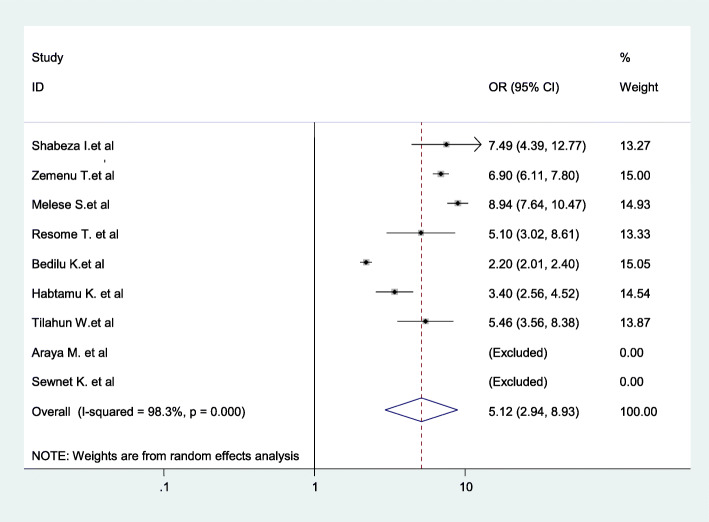
Association between walking distance to the nearest health center and childbirth at home in Ethiopia

Moreover, the odds of childbirth at home were 5.12 times higher among women who lived more than 2 hours walking distance to the nearest health center compared to those women who lived within 1 hour of the nearest center (AOR = 5.12, 95% CI: 2.94–8.93) (Fig. 14).

## Discussion

In this review, forty (40) studies comprising a total of 71, 724 participants were analyzed to estimate the best available evidence for the prevalence and factors associated with childbirth at home in Ethiopia. Accordingly, the pooled prevalence of childbirth at home was 66.7% (95%CI: 61.56–71.92). The result is in line with a study extracted from the 2016 Ethiopia Demographic and Health Survey accounting for 67.2% of mothers giving birth at home [90]. However, the result is higher than the study in Nepal [91] 41.9%, Senegal [69] 43.5%, and Guinea-Bissau [92] 61.2%, quite possibly due to difference in the availability of transportation, distance from the health facility, health literacy, wealth index, access to media like listening to radio/watching TV, mothers’ level of education, and knowledge of birth preparedness and complication readiness plan. As per the authors’ knowledge, this is the first systematic review and meta-analysis in Ethiopia to estimate the pooled prevalence and determinants of home childbirth. The result of this systematic review has a supreme importance to reduce home childbirth, to promote institutional delivery and skill birth attendants to reduce maternal and newborn complications. It also suggests the possible strategies to reduce home childbirth, the review can have clinical importance and potential policy response for health care systems. Therefore, the Ministry of Health and other stakeholders should continue the effort to decrease home childbirth through access to healthcare services, strengthen the coverage of antenatal care visits, promoting birth preparedness and complication readiness plan. and also a challenging duty that needs collaborative efforts from multi-sectored dimensions [93, 94]. The ministry of education could also strengthen girls’ empowerment through education that can promote health seeking behavior, decision making capacity, to enable them comprehend better about the potential risk associated with home childbirth and have a better idea about service availability [1, 95].

Even tough, home childbirth is unacceptably high, we found an overall decrease of childbirth at home in later researches (conducted between 2017 & 2018) (55.7%; 95%CI: 41.15–70.27) as compared to earlier surveys (conducted between 2012 & 2014) (69.3%; 95%CI: 59.68–78.84). The possible reason might be the establishment of maternity waiting homes (MWH) as a part of a strategy to improve access to skilled care by bringing pregnant women physically close to health facilities [96], and launch of Health Extension Program to provide health education about maternal and child health in households and in communities that promote healthy behaviors and utilization of health facilities during pregnancy and childbirth [97], which in turn decrease home childbirth.

Our study findings revealed women who did not pursuing ANC visits during pregnancy at all and had 1–3 ANC visits only were more likely to give birth at home as compared with those who had 4 or more antenatal care during pregnancy. ANC is the most favorable contact point for mothers to get more information about the risks and problems they may encounter during delivery. The World Health Organization (WHO) recommends that women without complications should have at least four antenatal visits, the first of which should take place during the first trimester [87]. Studies from Kathmandu, Nepal, and Malawi showed a strong connection between no/fewer than four ANC visits and home delivery [28, 98]. Additionally, this finding is consistent with studies conducted in Eretria [99], Bhutan [100], and Senegal [69]. The possible reason might be women who had no/few ANC follow-up might be less aware of birth preparedness and complication readiness plan, danger signs of pregnancy, when to visit health facilities, and the danger of giving childbirth at home which increases the probability of home childbirth. Additionally, mothers who did not receive antenatal care during pregnancy may not have adequate knowledge about institutional services for her and newborn, which hinder them to visit a health facility during childbirth.

Place of women’s residence, the rural residency was significantly associated with home childbirth. This finding is consistent with a study in Nigeria [101], Guinea-Bissau [102], Pakistan [103], and Bhutan [104]. The possible explanations might be rural residents, namely, less proportion of educated mothers, poor knowledge of institutional delivery services, less antenatal care follow-up service, less availability of health care services nearby, and poor access to information than urban mothers. Moreover, factors like cultural and religious beliefs might have been stronger in the rural area which promotes home childbirth.

Moreover, women who had poor knowledge of obstetric complications were more likely to give birth at home as compared to women who had good knowledge of obstetric complications. This finding is supported by a study done in Sub-Saharan Africa [105]. The possible explanation might be knowledge of obstetric complications is essential for early recognition of the problem, appropriate, and timely utilization of institutional delivery services. Thus, women who do not have good knowledge about obstetric complications, tend to give childbirth at home. Moreover, the lack of information on the warning signs of complications during pregnancy, parturition, and postpartum period hampers women’s ability to partake fully in institutional delivery services.

Women who had no birth a preparedness and complication readiness plan were more likely to give birth at home as compared to women who had a preparedness and complication readiness plans. This finding is supported by studies in Senegal [106], Uganda [107], and Tanzania [108]. The possible explanation might be women who have no plan for childbirth and not prepared for complications, allowing women to give birth at home and less involved in the management of pregnancy.

This review also revealed a significant association between women’s’ educational status and home delivery. Women who had no formal education and had greater odds of having a home childbirth than those who had formal or higher education. This is in line with studies in Nepal [109], and Uganda [110]. This might be because educated women comprehend better about the potential risk associated with home childbirth and have a better idea about service availability. Moreover, uneducated women might have poor decision-making capacity of seeking maternal health care services which prone them to give birth at home.

Our findings showed that women who had no access to media (radio or television) were more likely to give birth at home as compared to women who had access to media. The result is consistent with the study in Ghana [111]. Women who have no radio and television are more likely to have poor knowledge about pregnancy and labor, including danger signs and complications, and the need for professional help which lowers women’s health-seeking behavior and increased home childbirth practices.

The results of this systematic review showed a significant association between walking distance to the nearest health facility and home delivery. Women who lived greater than 2 hours walking time (distance) to the nearest health center as compared to those less than 1 hour walking time had considerably higher odds of childbirth at home. This finding is in line with studies in Kathmandu, Kaoma, and Zambia [112–114]. The possible reason might be women facing long walks may be particularly unwilling to consider travel to health facilities during the critical hours preceding childbirth. In a country where 85% of the population resides in rural areas with poor infrastructure and inaccessible roads, many Ethiopian women are simply too far from a facility to consider institutional delivery.

### Limitation

Since it is the first systematic review and meta-analysis, it is taken as strength. The included articles were restricted to the English language only; this is a limitation of the study as it missed studies published in local languages. This review has not registered online.

## Conclusion

Even though the government tried to lower the rate of home childbirth by promoting the importance of institutional delivery, the rate of home childbirth is stagnant so far in Ethiopia. Being from a rural area, we have no formal education, not pursuing ANC visits at all, having 1–3 ANC visits only, no media access, poor knowledge of obstetric complications, no birth preparedness and complication readiness plan, and walking time greater than 2 hours to reach the nearest health facility increased the probability of home childbirth in Ethiopia. Therefore, to improve the health-seeking behavior of women, it is better to improve the awareness of the community, families, and women about obstetric danger signs through factor-specific interventions. Counseling services about institutional delivery and obstetric complications during ANC visits should be strengthened. Community-based health information, the enlightenment of groups of women, and use of electronic media to disseminate health information could help women and the community at large to have a better awareness of institutional delivery service, the importance of ANC following up, and obstetric danger signs of pregnancy in a more timely fashion which in turn decrease home delivery practice. Moreover, expanding media access about maternal health, improving women’s educational status, creating access to health facilities, and improve infrastructure like transportation systems including ambulance service would decrease home childbirth practices.

### Availability of data and materials

All related data has been presented within the manuscript. The dataset supporting the conclusions of this article is available from the authors on request.

## Data Availability

The data sets generated during the current study are available from corresponding author on reasonable request.

## Abbreviations

ANC: Antenatal care
AOR: Adjusted Odd Ratio
CI: Confidence interval
EDHS: Ethiopia Demographic and Health Survey
JBI: Joanna Briggs Institute
OR: Odds Ratio
PRISMA: Preferred Reporting Items for Systematic Review and Meta-Analysis statement
SNNPR: Southern Nation Nationality and Peoples Representatives
